# Novel potentially pathogenic variants detected in genes causing intellectual disability and epilepsy in Polish families

**DOI:** 10.1007/s10048-023-00724-w

**Published:** 2023-07-05

**Authors:** S. Skoczylas, P. Jakiel, T. Płoszaj, K. Gadzalska, M. Borowiec, A. Pastorczak, H. Moczulska, M. Malarska, A. Eckersdorf-Mastalerz, E. Budzyńska, A. Zmysłowska

**Affiliations:** 1Department of Clinical Genetics, Medical University of Lodz, Lodz, Poland

**Keywords:** Next-generation sequencing, Pathogenic variants, Intellectual disability, Epilepsy

## Abstract

**Background:**

Intellectual disability (ID) affects 1–3% of the world population. The number of genes whose dysfunctions cause intellectual disability is increasing. In addition, new gene associations are constantly being discovered, as well as specific phenotypic features for already identified genetic alterations are being described. The aim of our study was to search for pathogenic variants in genes responsible for moderate to severe intellectual disability and epilepsy, using a panel of targeted next-generation sequencing (tNGS) for diagnosis.

**Methods:**

The group of 73 patients (ID, *n*=32; epilepsy, *n*=21; ID and epilepsy, *n*=18) was enrolled in the nucleus DNA (nuDNA) study using a tNGS panel (Agilent Technologies, USA). In addition, high coverage mitochondrial DNA (mtDNA) was extracted from the tNGS data for 54 patients.

**Results:**

Fifty-two rare nuDNA variants, as well as 10 rare and 1 novel mtDNA variants, were found in patients in the study group. The 10 most damaging nuDNA variants were subjected to a detailed clinical analysis. Finally, 7 nuDNA and 1 mtDNA were found to be the cause of the disease.

**Conclusions:**

This shows that still a very large proportion of patients remain undiagnosed and may require further testing. The reason for the negative results of our analysis may be a non-genetic cause of the observed phenotypes or failure to detect the causative variant in the genome. In addition, the study clearly shows that analysis of the mtDNA genome is clinically relevant, as approximately 1% of patients with ID may have pathogenic variant in mitochondrial DNA.

**Supplementary Information:**

The online version contains supplementary material available at 10.1007/s10048-023-00724-w.

## Introduction

Intellectual disability (ID) affects 1–3% of the world’s population [1]. It is the most common neurodevelopmental disorder characterized by a reduction in intellectual functioning and adaptive behaviour that originates in the child’s developmental period. Depending on the severity of the intellectual impairment, ID is classified as mild, moderate, and severe, using individually standardized tests. Most patients are diagnosed with mild ID [1, 2]. In addition, epilepsy is one of the most common comorbidities with ID and can affect its course, especially if seizures begin at a very young age and cannot be well controlled with standard antiepileptic drugs. Approximately 22% of patients with ID have seizures, and the prevalence of epilepsy increases with the severity of ID [3]. Genetic testing is increasingly important in identifying the causes of epilepsy [4]. The DisGeNET database (https://www.disgenet.org/) has so far listed 1215 genes involved in the disease [5]. Several of these have been identified as being directly involved in epilepsy [6]. Interestingly, also pathogenic variants in mitochondrial DNA (mtDNA) can also cause ID and/or seizures. However, in patients with pathogenic mtDNA variants, the clinical picture is heterogeneous due to the presence of a mixture of mutant and wild-type mtDNA, called heteroplasmy, in various cells and tissues. The level of heteroplasmy plays a key role in determining the phenotype [7]. The aim of our study was to look for pathogenic variants in genes responsible for the development of ID and/or epilepsy in the nucleus DNA (nuDNA) and pathogenic mtDNA variants using the targeted next-generation sequencing (tNGS) technique.

## Material and methods

### Clinical characteristic of patients

The study recruited 73 patients (50.7% men and 49.3% women) with ID with or without epilepsy who were referred to the Clinical Genetics Clinic of the Department of Clinical Genetics at the Medical University of Lodz in 2019–2021 and underwent pedigree and medical examination. The study group included 34 children with ID/developmental delay (47%), 21 with epilepsy (29%), and 19 patients with both conditions (24%). The mean age was 13±12 years. All patients who participated in this study were directed in the tNGS panel described below. In addition, we extracted mtDNA data for only 54 patients from this panel due to low coverage for other patients. Finally, we found a genotype-phenotype correlation in 16 patients and these were subjected to a more extensive review. Clinical and genetic characteristics of these patients with identified pathogenic gene variants are described in Table 1. Analysis of allele segregation in the family using the Sanger method was possible and was performed for 10 families and is shown in Supplementary Figure 1. The parents of patients on the first visit in our Clinic declared that they are biological parents, and it was not checked.

**Table 1:** Clinical and genetic characteristics of patients with identified pathogenic gene variants

Patient ID	Age at the time of the study (years)	Gender	Level of intellectual disability (age of examination)	Epilepsy	mtDNA	Variant	Pattern of inherited	gnomAD allele frequency nuDNA	Details of ACMG rating of nuDNA	ClinVar nuDNA	ACMG classification nuDNA
Patient I	9	Female	Severe (5 years)	No	m.8463A>G	KIF1A:c.3826C>T	Paternal pattern	0.000004136	PVS1, PM2, PP5	1× pathogenic	Pathogenic
Patient II	1,5	Female	No	First episode at 6 months	_	KCNQ2:c.641G>A	De novo	0	PS2, PM1, PM5, PP3, PP5, PM2	1× pathogenic	Pathogenic
Patient III	7	Female	Moderate	Yes/sleep seizures	_	SCN3A:694+2T>G	De novo	0	PS2, PVS1, PM2	_	Likely pathogenic
Patient IV	9	Female	Moderate (9 years)	Yes	m.5591G>A and m.9445G>A	SCN8A:c.2507A>T	Maternal pattern	0	PM1, PM2, PP3, PP2	_	Likely pathogenic
Patient V	28	Male	Yes	No	_	KIF1A:c.3826C>T	Patternal pattern	0.000004136	PVS1, PM2, PP5	1× pathogenic	Pathogenic
Patient VI	5	Male	Yes	No	m.7947A>G	_	Not investigated	_	_	_	_
Patient VII	5	Female	Yes	Yes	_	SYNE1:c.25922G>A	Maternal pattern	0.0001167	PM2, PP5, BP1, BP4	3× VUS	VUS
Patient VIII	3	Female	Severe (14 years)	No	_	SYNGAP1:c.2793_2794delCT	De novo	0	PS2, PVS1, PP5, PM2	2× pathogenic	Pathogenic
Patient IX	19	Female	Yes	No	_	SYNGAP1:c.427C>T	De novo	0	PS2, PVS1, PP5, PM2	9× pathogenic	Pathogenic
Patient X	13	Female	No	Focal seizures	_	TBC1D24:c.32A>G	Paternal pattern	0	PP3, PM2, PM1	_	VUS
Patient XI	4	Male	No	Yes	_	SCN1A:c.671T>C	Not investigated	0	PM1, PP3, PM5, PP5, PM2	1× likely pathogenic	Pathogenic
Patient XII	9	Female	Yes	No	m.5521G>A	_	Not investigated	_	_	_	_
Patient XIII	7	Female	Moderate (7 years)	Yes	_	PIGV:c.1022C>A homo	Not investigated	0.0001131	PP5, PM5, PP3, PM2	1× likely pathogenic, 10× pathogenic	Pathogenic
Patient XIV	3	Female	Yes	No	_	SCN1A:c.4793A>T	Not investigated	0.000008	PM1, PP3, PM2	4× VUS	Likely pathogenic
Patient XV	14	Female	Mild/moderate (14 years)	No	_	PIGV:c.1022C>A	Not investigated	0.0001131	PP5, PM5, PP3, PM2	1× likely pathogenic, 10× pathogenic	Pathogenic
Patient XVI	2	Female	No	Drug-resistant epilepsy	_	PIGV:c.1022C>A	Not investigated	0.0001131	PP5, PM5, PP3, PM2	1× likely pathogenic, 10× pathogenic	Pathogenic

### Library preparation

All patients were directed to tNGS Sure SelectQXT Panel (Agilent, Santa Clara, USA) with selected 132 genes tightly associated with the above-mentioned disease and mtDNA. The list of genes is described in Supplementary Table 1. DNA was extracted from peripheral blood using Maxwell® RSC Instrument (Maxwell® RSC Blood DNA Kit, Promega, Madison, USA). The library was prepared using the Agilent SureSelectQXT Target Enrichment protocol with a custom gene panel according to the manufacturer’s instructions. The paired-end sequencing was performed on a NextSeq550 System, Illumina (2×150 bp).

### Bioinformatics analysis

Bioinformatic analysis was performed using the local instance of Galaxy (https://usegalaxy.org/) [8] by mapping the raw FASTQ file to the GRCh38/hg38 reference genome using Map with BWA-MEM algorithm [9] and then removing duplicates using Picard tools [10]. Analysis of target regions and mapping quality was performed using BEDTools [11] (coverage statistics, sequencing depth>30, quality>1500). We used FreeBayes algorithm for variant calling [12], VCF files were merged with the VCFcombine tool [13], and functional annotation was performed using VEP (Variant Effect Predictor) [14] and wANNOVAR [15], and GEMINI [16] was applied (supports only GRCh37/hg19). Furthermore, we performed genome association study for our variants compared to The Thousand Polish Genomes database [17] by filtering BED files, excluding variants that are not present in the healthy Polish population and in vice versa. Mitochondrial sequences were analysed using the bioinformatic pipeline from Płoszaj et al. [18]. Allele frequencies and other calculations on the VCFcombine file were carried out using the program vcflib [13] to conduct target-wide association studies using qqman package for visualizing GWAS [19] in R studio [20]. Statistical analyses were performed with MedCalc for Windows, version 19.4 (MedCalc Software, Ostend, Belgium).

### Variant selection criteria

The variants of the studied genes were filtered for frequency in the GnomAD database for the European population below 0.01 [21]. They were then filtered for their damaging potential using Mutation Taster [22], EIGEN [23], SIFT (Sim et al., 2012), and CADD [24], and the conserved ratio was predicted using GERP [25]. The American College of Medical Genetics and Genomics (ACMG) classification [26] was also applied. In our study, only nonsynonymous variants in the coding region were considered as missense, frameshift, and stop gain mutations. The sequencing quality of the indicated variants had to meet the minimum coverage requirement of 120× coverage and allelic balance ~50%. Patients’ phenotypes were linked to the variants using the DisGeNET platform (https://www.disgenet.org/) and the OMIM database (https://www.omim.org/) [27]. We used specific selection criteria for VUS; we considered variants only if almost all or all prediction algorithms mentioned above predicted a deleterious effect of variants and a given variant was located in a conserved region. A wide association study of the data from our 73 patents compared with the Thousand Polish Genomes database filtered by bed of 132 genes from the tNGS panel was described in the Manhattan plot. Mitochondrial DNA analysis required a slightly different approach; we searched for confirmed pathogenic mitochondrial variants in the MitoPhen database (https://www.mitophen.org/) [28] and MITOMAP (https://www.mitomap.org/MITOMAP) [29]. If our variants were absent, we filtered the frequency of homoplasmic and heteroplasmic variants in the HelixMTdb database [30]. Subsequent analyses included verification of the prediction score for no confirmed pathogenic variants by APOGEE [31] for protein-coding genes and for variants in rRNA, MitoTIP [32].

### tNGS variant confirmation

All phenotype-associated variants were confirmed by Sanger sequencing. DNA fragments of the reference sequence, including the coordinates of specific variants, were downloaded from the UCSC database (genome.ucsc.edu) [33], and then primers were designed using the online tool Primer3 (primer3.ut.ee) [34]. PCR amplification was performed using KAPA HiFi HotStart ReadyMix (Roche, Basel, Switzerland) 12.5 μl, forward and reverse primers 0.6/0.6 μl, water 11.3 μl, and 1 μl DNA. The thermocycler programme included an initial denaturation at 95 °C for 3 min, followed by 30 cycles of denaturation at 98 °C for 20 s, annealing at 60–64 °C (depending on the primer) for 15 s, and extension at 72 °C for 15 s, followed by a final extension at 72 °C for 1 min. For the GC capture fragment, we added DMSO to obtain a 5% concentration. We used classical agarose gel to check the PCR products, and then purification of the PCR products was performed using AMPure XP magnetic beads (Beckman Coulter, California, USA), and then for labelling we used BrightDye™ Terminator Cycle Sequencing Kit v3.1 (NimaGen, Nijmegen, Netherlands). The BigDye® XterminatorTM Purification Kit (Applied Biosystems, Massachusetts, USA) was used for a second purification step following the standard protocol. The 3500 Genetic Analyzer platform (Applied Biosystems, Massachusetts, USA) was used for Sanger sequencing. Collected sequences were opened with Chromas 2.6.6 and then we used BLAST: Basic Local Alignment Search Tool on the NCBI (National Center for Biotechnology Information) website (https://blast.ncbi.nlm.nih.gov/Blast.cgi) to obtain information about variants in the sequence [35].

## Results

**Fig. 1:**
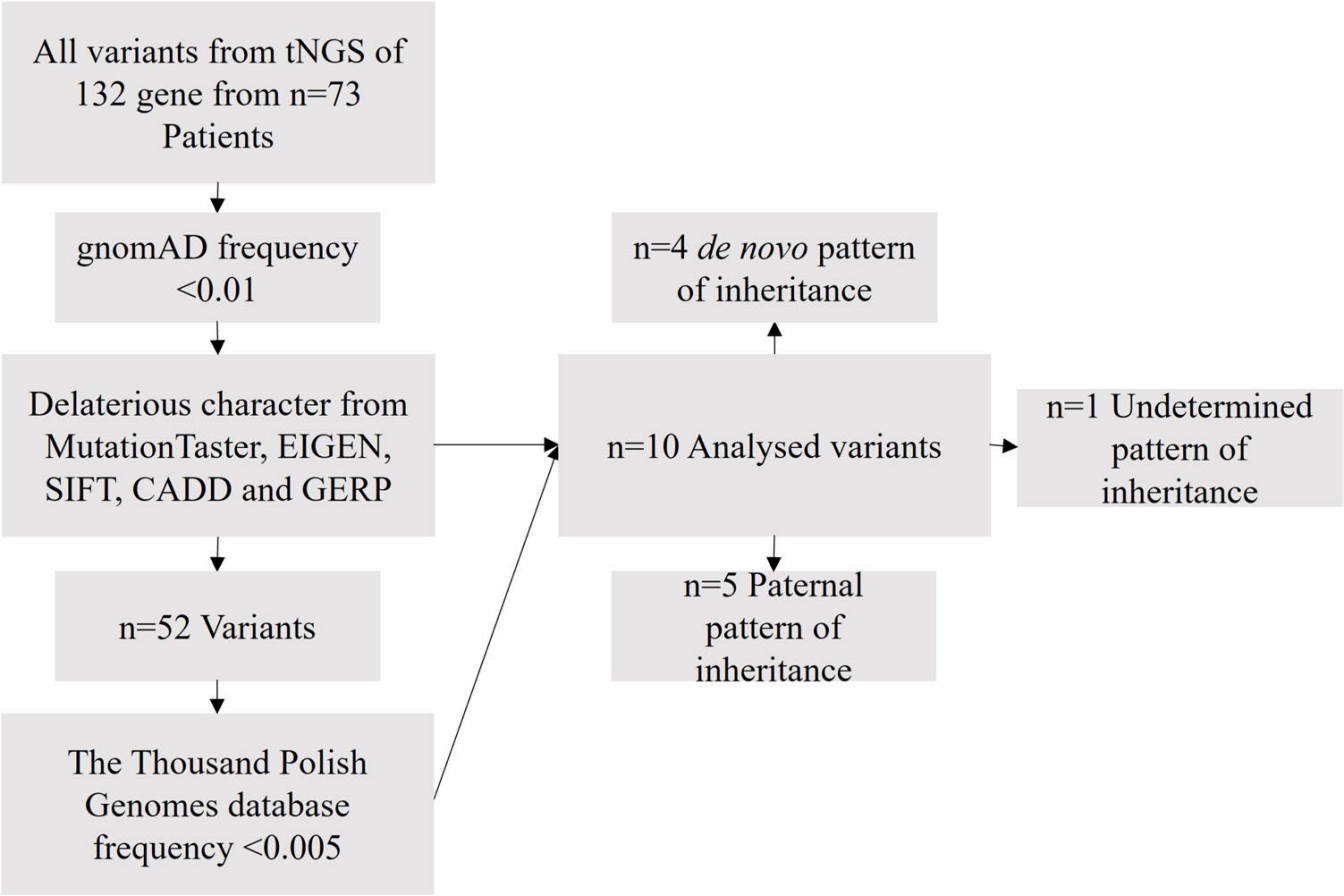
Workflow for chose the most deleterious and rarest variants

The first step of the analysis was a wide association study of the data from our 73 patients compared with The Thousand Polish Genomes database filter by BED of 132 genes from tNGS panel. Moreover, in a plot of signal intensity (Manhattan plot) relative to genomic position, *P* values (minus log-transformed) are shown in Supplementary Figure 2. It is not possible to clearly identify significant variants above the threshold from the results of a wide association study. For one reason, the study group was too small to be reliably representative of the population. The second reason is probably not enough genes in the tNGS panel. Finally, we selected 52 rare variants if the nuDNA had the following ACMG classification: uncertain significance (VUS) *n*=30, likely pathogenic *n*=10, and pathogenic *n*=12. We then selected the rarest and most deleterious variants. Finally, 10 families were analysed in detail. The workflow for the selection of these variants is described in Fig. 1.

We identified four de novo variants: *KCNQ* p.Arg214Gln, *SCN3A*:c.694+2T>G, *SYNGAP1* p.Phe932fs, *SYNGAP1* p.Arg143Ter; five paternally inherited variants: *KIF1A* p.Arg1276Ter, *PAFAH1B1* p.Val396Ile, *PIGV* p.Ala341Glu, *SCN8A* p.Asp836Val, *TBC1D24* p.Asp11Gly; and one variant of unknown inheritance pattern: *SCN1A* p.Leu224Ser. The full list of selected variants is available in Supplementary Table 2. Supplementary Table 3 contains detailed statistical information about odds ratio. The patients’ phenotypes are described in more detail in the Supplementary file.

**Fig. 2:**
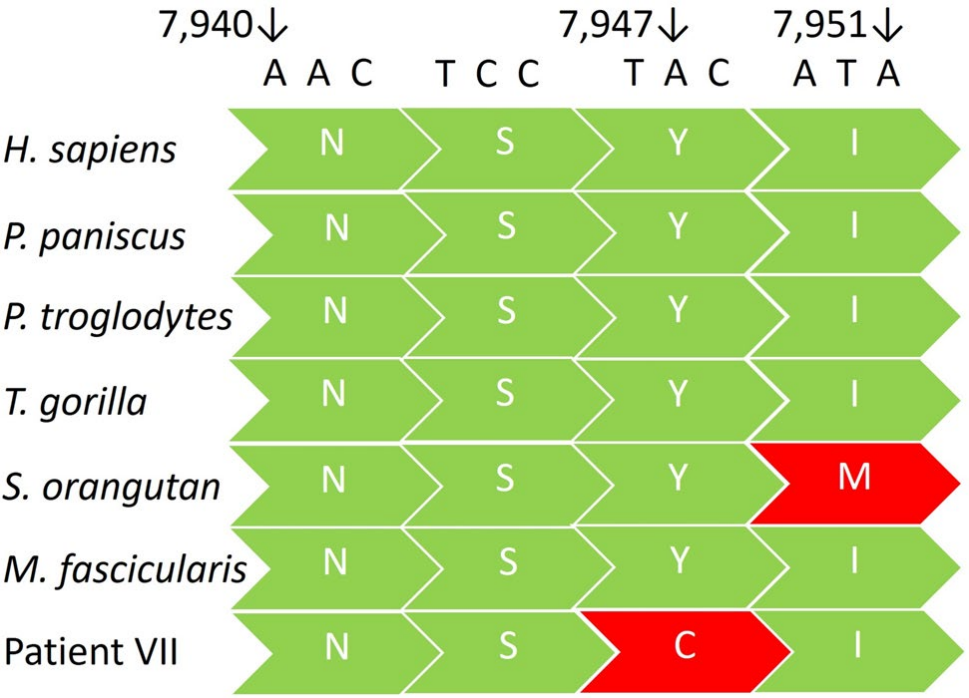
MT-CO2 gene position 7947 in different species and patient VI

In addition, we identified 11 rare heteroplasmic mitochondrial variants, small deletions and insertions were not clinically relevant according to current knowledge. In 54 patients with ID and/or epilepsy, we found one new heteroplasmic variant m.7947A>G, *MT-CO2*: c.362A>G (p.Tyr121Cys) in a female with 1.9% heteroplasmy in peripheral blood leukocytes. This results in a change from the conservative tyrosine to cysteine. The *MT-CO2* gene is highly conserved, particularly in *Pan paniscus* and *P. troglodytes* [36]. Figure 2 provides information on position m.7947 and amino acids in selected primates.

Prediction programmes revealed the pathogenic nature of the m.7947A>G variant which is described in Supplementary Table 5. Moreover, m. 5521G>A variant was detected; other variants have ambiguous classification. All rare mitochondrial variants detected in our cohort are described in Table 2. Variability for every 100 base pairs is shown in Supplementary Figure 5. Haplogroups were checked using Mitoverse (mtDNA-Server) to control for contamination [37].

**Table 2:** Rare mitochondrial variants detected in our cohort

Nucleotide position	Locus	Nucleotide (AA change)	Variant level (%)	HelixMTdb base-number homoplasmic vs heteroplasmic	Conservation	APOGEE
3209	*MT-RNR2,MT-RNR3*	A-G (rRNA)	1.74	10/1	62.22%	NA
5521	*MT-TW*	G-A (tRNA Trp)	1	0/1	93.33%	Pathogenic
5536	*MT-TW*	A-G (tRNA)	1.36	1/1	93.33%	MitoTIP:24.90% likely benign HmtVar: 0.05
5591	*MT-TA*	G-A (tRNA)	1	0/1	91.11%	MitoTIP:68.40% possibly pathogenic HmtVar: 1
7452	*MT-TS1*	A-G (tRNA)	1.5	0/0	95.56%	MitoTIP:59.80% possibly pathogenic HmtVar:0.05
7947	*MT-CO2*	A-G (Y121C)	1.9	0/0	NA	NA
8463	*MT-ATP8*	A-G (Y33C)	99	55/1	53.33%	P (0.53) possibly pathogenic
9166	*MT-ATP6*	T-C (F214L)	1.4	19/0	100.00%	N (0.4) possibly benign
9445	*MT-CO3*	G-A (R80Q)	0.8	0/11	100.00%	N (0.39) possibly benign
10405	*MT-TR*	T-C (tRNA)	2.3	0/4	77.78%	MitoTIP:61.00% possibly pathogenic HmtVar:0.05
11460	*MT-ND4*	T-C (V234A)	15.3	1/1	46.67%	N (0.39) possibly benign

## Discussion

In this study, we identified 52 nuDNA and 11 heteroplasmic mtDNA variants; all of them are rare. Finally, 8 nuDNA and 1 mtDNA were found to be the cause of the disease. Variants for which we have confirmed their pathogenic character with other work included *KCNQ2*:c.641G>A (p.Arg214Gln) de novo variant detected in a heterozygous female (patient II) causing an autosomal dominant disorder as confirmed by Fang Z et al. [38]. Further variant was *PIGV*:c.1022C>A (p.Ala341Glu) detected in one homozygous female (patient XIII) and two heterozygous females (patient XV and patient XVI), and classified in ClinVar as a cause of hyperphosphatasia with intellectual disability syndrome 1 (MIM #239300). Finally, this variant was only recognised as a cause of disease in homozygous patients. In addition, two de novo heterozygous variants in *SYNGAP1* gene c.2793_2794delCT (p.Phe932fs) and c.427C>T (p.Arg143Ter) were detected in 2 females (patient VIII) and (patient IX) respectively. The first one is submitted in ClinVar by single submitter with similar symptoms to our case. The second one was described for example by Mignot C et al. as a cause of disease [39].

The remaining 7 variants were not described in the literature until 2022. The first one is *KIF1A*:c.3826C>T (p.Arg1276Ter) variant detected in one heterozygous female (patient I). Heterozygous pathogenic variants in the *KIF1A* gene can cause NESCAV syndrome (MIM #614255) or spastic paraplegia 30, autosomal dominant (MIM #610357). In addition, missense variants can also cause an autosomal recessive spastic paraplegia (MIM #610357). Our patient suffered from severe intellectual disability and delay in psychomotor development, with concomitant abnormal EEG, and the proband has two asymptomatic brothers (not investigated) and asymptomatic parents. This variant has a paternal model of inheritance, which suggests no causative character of this variant, since family members carrying this variant have no symptoms and secondly in the GnomAD database 1 female participant carries this variant. On the other hand, in the clinical exome data from our laboratory, we have another family with a similar pattern of segregation (patient V, data unpublished). Both patients have development delay, intellectual disability, and long forehead in common. In this case, we consider incomplete penetrance of the variant as a cause of phenotype in patient. At present, we have classified the *KIF1A*:c.3826C>T variant as the cause of autosomal dominant disease.

The two heterozygous variants in *SCN1A* gene c.671T>C (p.Leu224Ser) and c.4793A>T (p. p.Tyr1598Phe) were detected in one male (patient XI) and in one female (patient XIV) respectively. Pathogenic variants in this gene cause a few autosomal dominant diseases (for example, MIM #619317 or #607208). For both cases, segregation analysis was not possible due to loss of contact with the patient’s parents. For the first one, phenotype of the proband is similar to the disease associated with pathogenic variants in the *SCN1A* gene and this variant is absent in gnomAD database and in The Thousand Polish Genomes database. The c.4793A>T was carried by 2 individuals in gnomAD database, and we recognised this variant as VUS.

Another is the *SCN3A*:c.694+2T>G de novo variant detected in one heterozygote female (patient III). Developmental and epileptic encephalopathy 62 (MIM #617938) and epilepsy, familial focal, with variable foci 4 (MIM #617935), are caused by a heterozygous mutation in the *SCN3A* gene. To date, no splice site pathogenic variant has been described in this gene. Moreover, this variant is absent in the gnomAD database and in The Thousand Polish Genomes database. Finally, we classified this variant as likely pathogenic.

In addition, the *SCN8A*:c.2507A>T (p.Asp836Val) variant was detected in one heterozygote female (patient IV). Cognitive impairment with or without cerebellar ataxia (MIM #614306) and developmental and epileptic encephalopathy 13 (MIM #614558) are autosomal dominant disorders. The patient’s mother who also carries the variant has mild intellectual disability. Two asymptomatic sisters of proband do not carry this variant. In addition, *SCN8A*:c.2507A>T variant is absent in gnomAD database and it is classified as VUS according to the ACMG classification. In our study, we recognised this variant as pathogenic due to segregation with the phenotype in family members and zero frequency in healthy population.

Moreover, the *SYNE1*:c.25922G>A (p.Arg8641Gln) variant was detected in a heterozygote female (patient VII). Emery-Dreyfuss muscular dystrophy 4 (MIM #612998) is an autosomal dominant disorder and spinocerebellar ataxia, autosomal recessive 8 (MIM #610743). We considered this variant because of its absence in The Thousand Polish Genomes. However, this variant has a maternal pattern of inheritance, and the mother of patient VII is asymptomatic. All these data finally allow us to classify the *SYNE1*:c.25922G>A variant as VUS.

The last nuDNA variant recognised as disease cause is *TBC1D24*:c.32A>G (p.Asp11Gly) heterozygous variant in female (patient X). Myoclonic epilepsy, infantile, familial or epilepsy, rolandic, with paroxysmal exercise-induce dystonia and writer’s cramp (MIM #605021, #608105), is an autosomal recessive disorder. Although Banuelos E. et al. have described families with dominant and recessive phenotypes of tonic-clonic and myoclonic epilepsy [40], our patient has a paternal inheritance pattern, but the proband’s father is asymptomatic. We have no information about other paternal family members. One author described a few compounds heterozygous for the *TBC1D24* gene. However, in one family in this paper (siblings carried c.32A>G + c.1008delT), the paternal grandmother and the paternal half-sister suffered from epilepsy. The father has c.32A>G but the rest of the family have not been tested [41]. The *TBC1D24*:c.32A>G variant is absent in The Thousand Polish Genomes database and gnomAD database. In our case and in the cases mentioned above, we could see that only females have some symptoms. Finally, we classified *TBC1D24*:c.32A>G as VUS.

In addition for mtDNA, we detected m.5521G>A variant in a female (patient XII) with 1% heteroplasmy in peripheral blood leukocytes. This variant is confirmed pathogenic variant by MITOMAP and MitoPhen. Our patient presents with psychomotor disorders, autism in children, abnormal changes in EEG, and decreased muscle tone. Further analysis was not possible due to lack of material for testing. Moreover, the m.7947A>G variant was detected in a female (patient VI). There are no confirmed pathogenic variants in the *MT-CO2* gene up to date according to MITOMAP and MitoPhen. Examination in other materials to check the heteroplasmy level of this variant and COX deficient is not possible due to lack of patient consent. Other rare variants in tRNA such as m.10405T>C and absent in HelixMTdb, m.7452A>G, were detected in 2 different patients. The m.10405T>C was reported as VUS by Wong Lee-Jun C. et al. [42]. We do not associate these 2 variants with the phenotype of the probands.

The presented data confirms the tNGS panel is a highly useful tool to identify genetic background of ID and/or epilepsy. However, there are still a significant number of patients who remain undiagnosed, and in such cases WES or WGS can play a crucial role.

## Conclusion

The obtained results indicate that the genetic basis of intellectual disability is very complex and heterogeneous. Of the 73 patients with suspected ID and/or epilepsy, clear causal variants were identified in only nine cases. This clearly shows that a very large proportion (89%) of patients remain undiagnosed and may need further testing. The reason for the negative results of our analysis may be a non-genetic cause of the observed phenotypes or failure to detect the causative variant in the genome. It should be noted that only the coding regions of genes known to be associated with ID were analysed. It is possible that an analysis including both the proband and his parents (the WGS trio) could provide a broader picture of the genetic basis of ID in these patients. It is also worth noting that mitochondrial genome analysis should be considered routine in ID patients, since in one case, a defect in the mitochondrial genome was likely the primary cause of the disease. In conclusion, it seems that the existing narrow tNGS panels are ineffective in explaining the genetic causes of ID and epilepsy in patients.

### Supplementary information

ESM 1 Figure 1 Allele segregation in 10 families. Table 1 Gene panel of tNGS. Figure 2 Manhattan plot for wide association study of 132 genes from tNGS panel. The blue line indicates the threshold at level -log_10_=5 and the red line at level -log_10_=7.5. Table 2 52 extremely rare variant from genome DNA. The variants discussed in the discussion are marked with grey colour. Table 3 Statistics for variants in study group and the thousand polish genomes. Table 4 Rare mitochondrial variants from 54 Patients. Table 5 Prediction score for m.7937A>G variant by Mitimpct 3D. Figure 5 The number of SNPs within 0.1 Kb window size. SNP density was plotted by http://www.bioinformatics.com.cn/plot_basic_SNP_density_by_CMplot_107_en, an online platform for data analysis and visualization. (DOCX 324 kb)

## Data Availability

The data generated during the current study are available from the corresponding author upon reasonable request.
