# Supplementary material for: Novel potentially pathogenic variants detected in genes causing intellectual disability and epilepsy in Polish families
Source: Neurogenetics. 2023 Jul 5;24(4):221–9. doi: 10.1007/s10048-023-00724-w (PMC10545623; doi:10.1007/s10048-023-00724-w)
Supplement: Supplementary file 1 — Figure 1 Allele segregation in 10 families. Table 1 Gene panel of tNGS. Figure 2 Manhattan plot for wide association study of 132 genes from tNGS panel. The blue line indicates the threshold at level -log10=5 and the red line at level -log10=7.5. Table 2 52 extremely rare variant from genome DNA. The variants discussed in the discussion are marked with grey colour. Table 3 Statistics for variants in study group and the thousand polish genomes. Table 4 Rare mitochondrial variants from 54 Patients. Table 5 Prediction score for m.7937A>G variant by Mitimpct 3D. Figure 5 The number of SNPs within 0.1 Kb window size. SNP density was plotted by http://www.bioinformatics.com.cn/plot_basic_SNP_density_by_CMplot_107_en, an online platform for data analysis and visualization. (DOCX 324 kb) [file 10048_2023_724_MOESM1_ESM.docx]

**Supplementary data**

Describe the patient's phenotypes:

Patient I: 9 years old, female, severe ID, global developmental delay, syncope, dizziness, coordination disorder, difficulties in walking upstairs

Patient II: 1.5 years old, female, epilepsy first episode at 6 months,

Patient III: 7 year old, female, moderate ID, sleep seizures, ADHD, bone exostoses above the knee joints

Patient IV: 9 year old, female, moderate intellectual disability, dysmorphic facial features, triangular face, long, protruding ears, prominent nose, neurotic disorders, microcephaly, immunodeficiency

Patient V: 28 year old, male, development delay, intellectual disability, long forehead

Patient VI: 5 year old, male, ID, developmental disorders in the family members, an autism diagnosis at age of 3 years

Patient VII: 5 year old, female, global developmental delay, muscle hypotrophy, autism, scoliosis, epilepsy

Patient VIII: 3 year old, female, hashimoto's disease, depression, absence of dysmorphic features, results indicating somating mosaicism aphasia, autistic behavior, microcephaly, dysmorphic facial features, deep-set eyes, almond shaped eyes, obesity, small hand and feet, unencumbered family history,

Patient IX: 19 year old, female, ID, developmental delay,

Patient X: 13 year old, female, focal seizures

Patient XI: 4 year old, male, epilepsy

Patient XII: 9 year old, female, ID

Patient XIII: 7 year old, female, moderate ID, epilepsy, hyperphosphatasia

Patient XIV: 3 year old, female, ID, global developmental delay, adrenal neuroblastoma in 6 months of life

Patient XV: 14 year old, female, ID mild/moderate (14 years), obesity, apnea, delayed gross motor development in first three years of life, High, narrow palate, microdontia, V-finger clinodactyly

Patient XVI: 2 year old, female, drug-resistant epilepsy


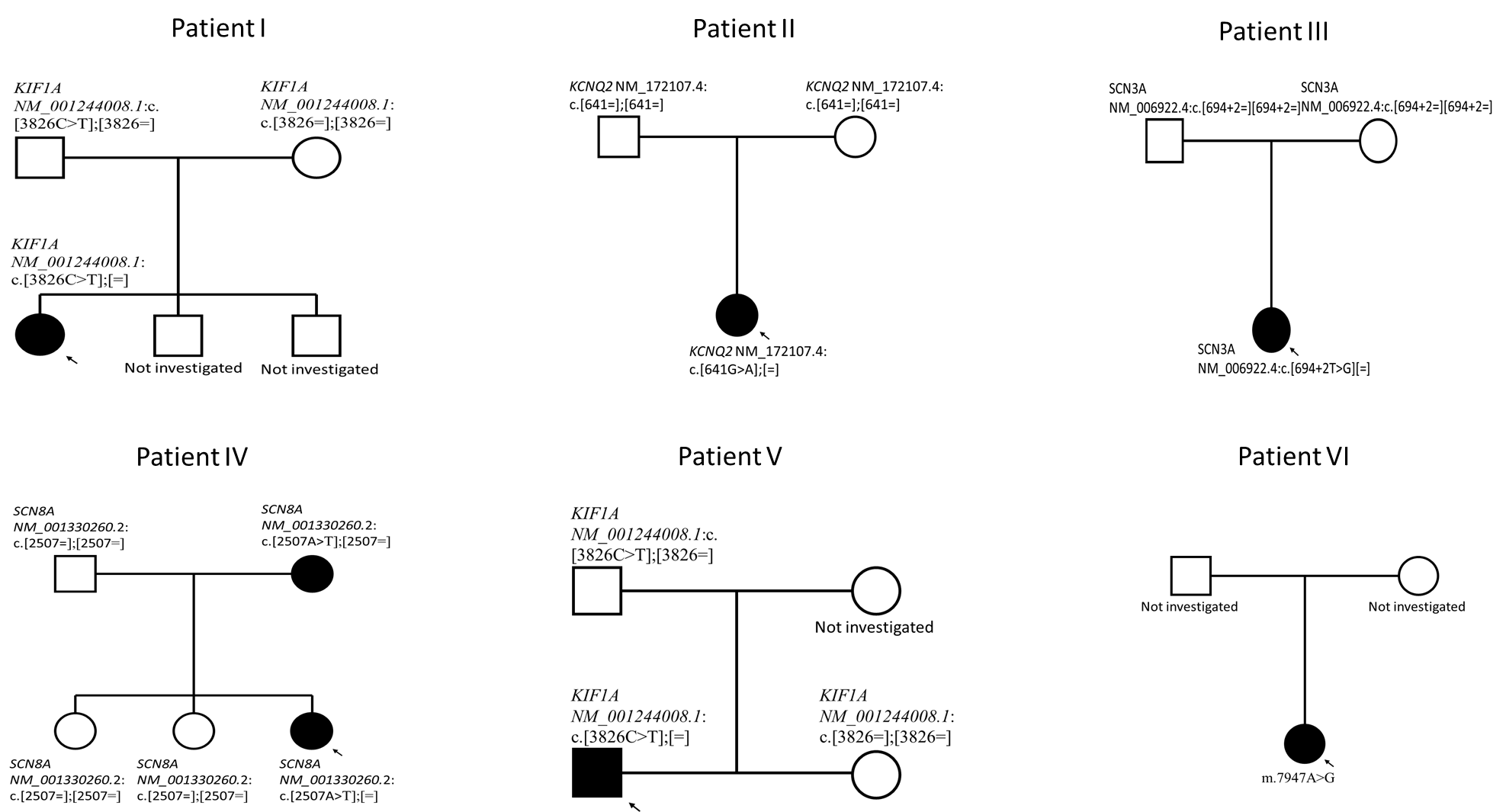


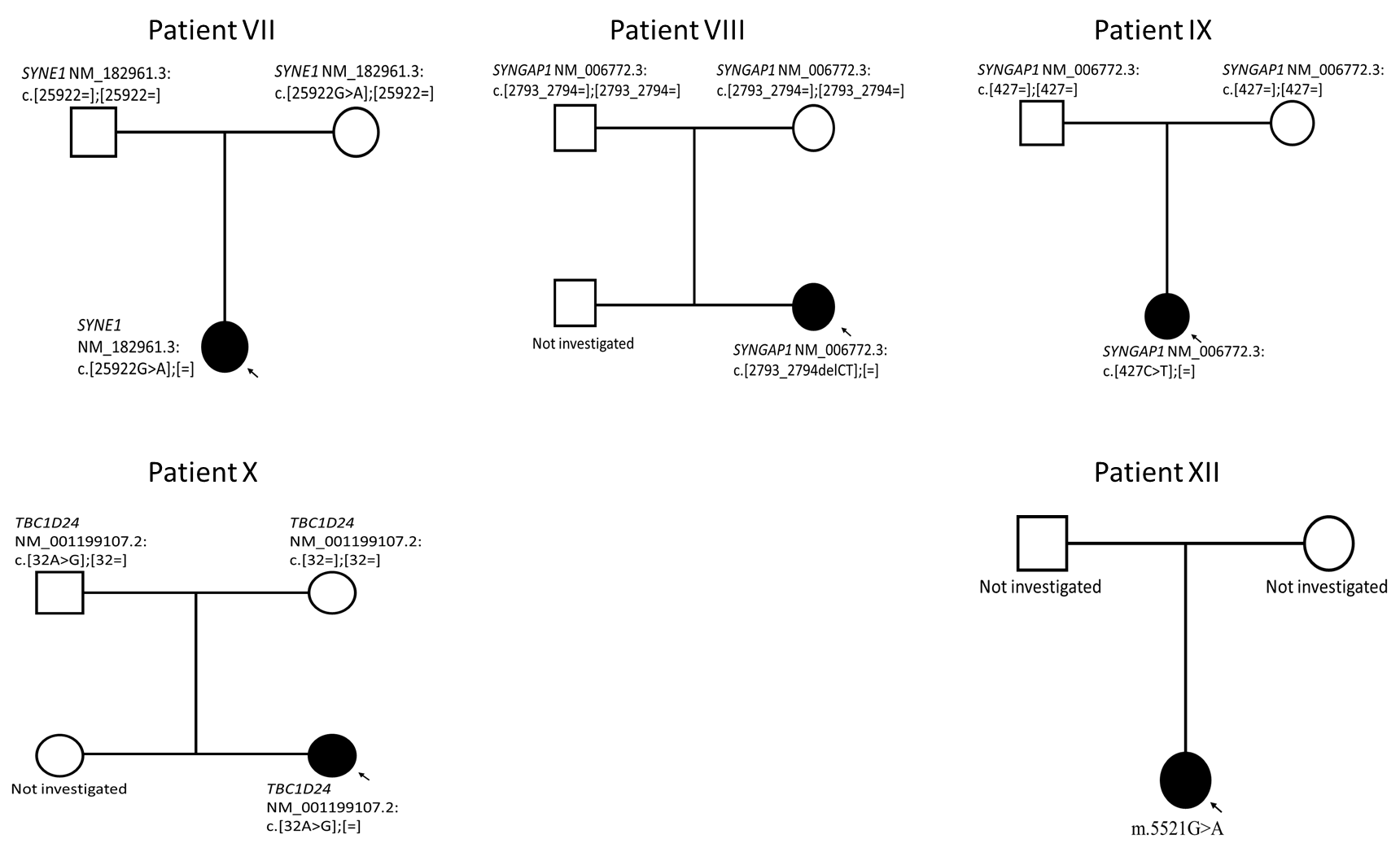


Figure 1 Allele segregation in 11 families.

Table 1 Gene panel of tNGS.

| **Gene** | **Chr** |
| --- | --- |
| *ABCC8* | chr11:17414528-17498333 |
| *ABCC9* | chr12:21953968-22089618 |
| *AIRE* | chr21:45705880-45717620 |
| *AKAP9* | chr7:91570404-91739483 |
| *ALMS1* | chr2:73612987-73836749 |
| *ANK2* | chr4:113825640-114302806 |
| *ARL6* | chr3:97486942-97516903 |
| *ATN1* | chr12:7043155-7050953 |
| *BAD* | chr11:64037513-64051850 |
| *BBS1* | chr11:66278121-66299518 |
| *BBS10* | chr12:76739583-76742148 |
| *BBS12* | chr4:123663038-123665190 |
| *BBS2* | chr16:56518663-56553784 |
| *BBS4* | chr15:72978559-73029938 |
| *BBS5* | chr2:170336054-170382216 |
| *BBS7* | chr4:122747005-122791478 |
| *BBS9* | chr7:33185855-33644848 |
| *BLK* | chr8:11400724-11421627 |
| *BRCA1* | chr17:41197685-41277212 |
| *BRCA2* | chr13:32890588-32972917 |
| *C2ORF86* | chr2:63349131-63815415 |
| *CACNA1C* | chr12:2080219-2800375 |
| *CACNA2D1* | chr7:81579698-82072785 |
| *CACNB2* | chr10:18429656-18828663 |
| *CALM1* | chr14:90863565-90871071 |
| *CASQ2* | chr1:116243852-116311172 |
| *CAV3* | chr3:8775553-8787563 |
| *CCDC28B* | chr1:32667527-32670859 |
| *CDK4* | chr12:58142298-58145510 |
| *CEL* | chr9:135944433-135947161 |
| *CEP290* | chr12:88442951-88535094 |
| *CHEK2* | chr22:29083875-29133281 |
| *CHGA* | chr14:93389695-93401239 |
| *CHGB* | chr20:5892270-5905705 |
| *CISD2* | chr4:103790232-103808597 |
| *COL1A1* | chr17:48262853-48278884 |
| *COL1A2* | chr7:94024334-94059715 |
| *CTNNA3* | chr10:67680078-69408538 |
| *DES* | chr2:220283175-220290722 |
| *DKK1* | chr10:54074185-54076577 |
| *DPP6* | chr7:153584759-154684200 |
| *DSC2* | chr18:28647971-28681944 |
| *DSG2* | chr18:29078205-29126716 |
| *DSP* | chr6:7542139-7586121 |
| *EIF2AK3* | chr2:88857244-88926802 |
| *ERN1* | chr17:62207167-62207399 |
| *FOXA1* | chr14:38060560-38064187 |
| *FOXA2* | chr20:22562478-22564926 |
| *FOXP3* | chrX:49107785-49115707 |
| *FXYD2* | chr11:117691377-117698738 |
| *G6PC2* | chr2:169757832-169764599 |
| *GATA4* | chr8:11565812-11615994 |
| *GATA6* | chr18:19751096-19780796 |
| *GCK* | chr7:44184725-44228562 |
| *GJA1* | chr6:121767984-121769152 |
| *GJA5* | chr1:147230260-147231356 |
| *GLIS3* | chr9:3828262-4286435 |
| *GLUD1* | chr10:88836323-88836423 |
| *GPD1L* | chr3:32148194-32207412 |
| *GRIK4* | chr11:120531018-120856979 |
| *HADH* | chr4:108910902-108955523 |
| *HCN4* | chr15:73614812-73660621 |
| *HNF1A* | chr12:121416419-121439005 |
| *HNF1B* | chr17:36047273-36104885 |
| *HNF4A* | chr20:42984435-43058315 |
| *IER3IP1* | chr18:44662779-44702658 |
| *INS* | chr11:2168832-2182211 |
| *ISL1* | chr5:50679496-50689454 |
| *JUP* | chr17:39775836-39928116 |
| *KCNA5* | chr12:5153304-5155165 |
| *KCND3* | chr1:112318689-112525358 |
| *KCNE1* | chr21:35821533-35821942 |
| *KCNE2* | chr21:35742768-35743159 |
| *KCNE3* | chr11:74168287-74168618 |
| *KCNE5* | chrX:108867811-108868259 |
| *KCNH2* | chr7:150642443-150675011 |
| *KCNJ11* | chr11:17408456-17410429 |
| *KCNJ2* | chr17:68171171-68172474 |
| *KCNJ5* | chr11:128781159-128786636 |
| *KCNJ8* | chr12:21918647-21926560 |
| *KCNQ1* | chr11:2465906-2869243 |
| *KLF11* | chr2:10183834-10192644 |
| *LMNA* | chr1:156084700-156109640 |
| *LMX1A* | chr1:165173107-165324806 |
| *LRP5* | chr11:68080173-68216548 |
| *MAFA* | chr8:144511505-144512586 |
| *MAFB* | chr20:39316509-39317500 |
| *MKS1* | chr17:56283430-56296882 |
| *MNX1* | chr7:156801305-156803054 |
| *NEUROD1* | chr2:182542507-182543597 |
| *NEUROG3* | chr10:71332145-71332809 |
| *NKX2-2* | chr20:21492551-21494317 |
| *NKX2-5* | chr5:172659562-172662096 |
| *NKX6-1* | chr4:85414432-85419391 |
| *NOS1AP* | chr1:162039958-162353331 |
| *NPPA* | chr1:11906056-11907751 |
| *ONECUT1* | chr15:53080673-53080721 |
| *PAX4* | chr7:127250896-127255992 |
| *PAX6* | chr11:31811472-31827969 |
| *PDX1* | chr13:28494266-28498848 |
| *PKP2* | chr12:32945348-33049675 |
| *PLN* | chr6:118880075-118880253 |
| *PRKAG2* | chr7:151254277-151573715 |
| *PTF1A* | chr10:23481450-23482845 |
| *RANGRF* | chr17:8192097-8193264 |
| *RFX6* | chr6:117198429-117252679 |
| *RYR2* | chr1:237205812-237995957 |
| *SCN1B* | chr19:35521715-35530615 |
| *SCN3B* | chr11:123504841-123524519 |
| *SCN4B* | chr11:118007732-118023398 |
| *SCN5A* | chr3:38591802-38674808 |
| *SDCCAG8* | chr1:243419466-243663097 |
| *SIRT1* | chr10:69644470-69676360 |
| *SLC16A1* | chr1:113456503-113471940 |
| *SLC19A2* | chr1:169435077-169455014 |
| *SLC2A2* | chr3:170715682-170744469 |
| *SLC9A3* | chr5:473484-524447 |
| *SLMAP* | chr3:57743369-57913125 |
| *SNTA1* | chr20:31996303-32031436 |
| *SOX2* | chr3:181430139-181431112 |
| *SOX9* | chr17:70117523-70120538 |
| *SREBF1* | chr17:17715059-17740141 |
| *SYT9* | chr11:7260089-7488041 |
| *TGFB3* | chr14:76425520-76447246 |
| *TMEM43* | chr3:14166684-14183305 |
| *TMEM67* | chr8:94781784-94781854 |
| *TRDN* | chr6:123539736-123957930 |
| *TRIM32* | chr9:119460012-119461993 |
| *TRPM4* | chr19:49661114-49714765 |
| *TTC8* | chr14:89291042-89343764 |
| *UCP2* | chr11:73686042-73689433 |
| *WFS1* | chr4:6279173-6304205 |


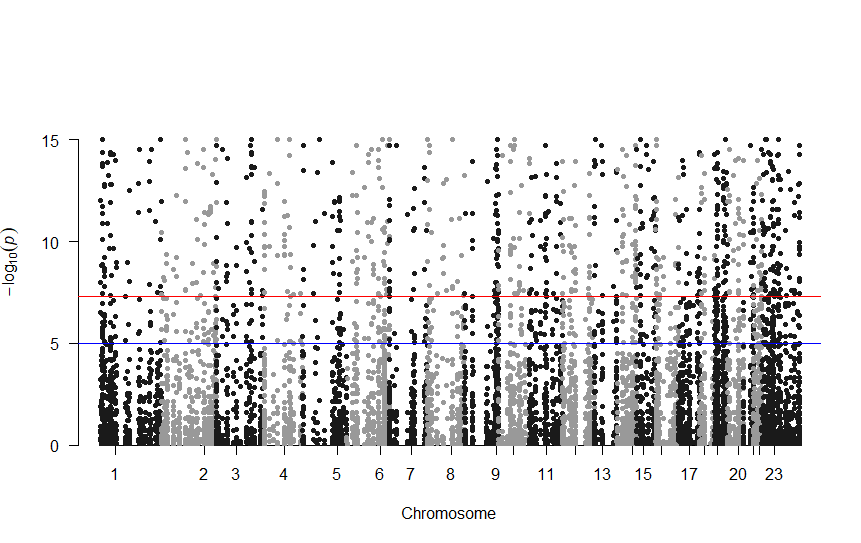


Figure 2 Manhattan plot for wide association study of 132 genes from tNGS panel. The blue line indicates the threshold at level -log_10_=5 and the red line at level -log_10_=7.5.

Table 2 52 extremely rare variant from genome DNA. The variants discussed in the discussion are marked with grey colour.

|  |  | |  |  | |  | |  | | |  |  | |  |  | |  | |  |  | |  |  |  |  |
| --- | --- | --- | --- | --- | --- | --- | --- | --- | --- | --- | --- | --- | --- | --- | --- | --- | --- | --- | --- | --- | --- | --- | --- | --- | --- |
| **chrom** | | **pos hg38** | | | **ref** | | **alt** | | ***gene*** | **transcript** | | | **codon change** | | | **aa change** | | **impact** | | | **rs ID** | | **ACMG** | **GnomAD v3.1.2 Allele Frequence** | **The Thousand Polish Genomes** |
| chr6 | | 73600406 | | | A | | G | | ***SLC17A5*** | NM_012434.4 | | | c.1295T>C | | | p.Phe432Ser | | missense_variant | | | rs200632199 | | **Likely Pathogenic** | 0.00027800 | 0.00371156 |
| chr8 | | 99853957 | | | A | | G | | ***VPS13B*** | NM_017890.4 | | | c.10643A>G | | | p.Tyr3548Cys | | missense_variant | | | rs398124326 | | **VUS** | 0.00017740 | 0.00371156 |
| chr1 | | 26795056 | | | C | | A | | ***PIGV*** | NM_001202554.1 | | | c.1022C>A | | | p.Ala341Glu | | missense_variant | | | rs139073416 | | **Pathogenic** | 0.000115 | 0.00318134 |
| chr11 | | 6617040 | | | G | | A | | ***TPP1*** | NM_000391.3 | | | c.622C>T | | | p.Arg208* | | stop_gained | | | rs119455955 | | **Pathogenic** | 0.00025640 | 0.00265111 |
| chr13 | | 51961868 | | | G | | A | | ***ATP7B*** | NM_000053.3 | | | c.1915C>T | | | p.His639Tyr | | missense_variant | | | rs200728096 | | **Likely Pathogenic** | 0.00009858 | 0.00212089 |
| chr13 | | 50945445 | | | G | | A | | ***RNASEH2B*** | NM_024570.4 | | | c.529G>A | | | p.Ala177Thr | | missense_variant | | | rs75184679 | | **Likely Pathogenic** | 0.00145200 | 0.00159067 |
| chr11 | | 134153147 | | | C | | T | | ***NCAPD3*** | NM_015261.2 | | | c.4381G>A | | | p.Asp1461Asn | | missense_variant | | | rs200132835 | | **VUS** | 0.00006571 | 0.00159067 |
| chr10 | | 95621105 | | | C | | T | | ***ALDH18A1*** | NM_001323413.1 | | | c.1393G>A | | | p.Glu465Lys | | missense_variant | | | rs757876226 | | **Likely Pathogenic** | 0.00003288 | 0.00159067 |
| chr8 | | 99720377 | | | A | | T | | ***VPS13B*** | NM_017890.4 | | | c.6765A>T | | | p.Leu2255Phe | | missense_variant | | | rs775506296 | | **VUS** | 0.00006571 | 0.00106045 |
| chr17 | | 623567 | | | C | | T | | ***VPS53*** | NM_001128159.2 | | | c.1082G>A | | | p.Arg361His | | missense_variant | | | rs766032255 | | **VUS** | 0.00004600 | 0.00106045 |
| chr13 | | 51974717 | | | A | | G | | ***ATP7B*** | NM_000053.3 | | | c.503T>C | | | p.Leu168Pro | | missense_variant | | | rs756237962 | | **Likely Pathogenic** | 0.00003945 | 0.00106045 |
| chr16 | | 70265641 | | | T | | C | | ***AARS*** | NM_001605.2 | | | c.1244A>G | | | p.Tyr415Cys | | missense_variant | | | rs373069396 | | **VUS** | 0.00002630 | 0.00106045 |
| chr13 | | 52011319 | | | CTG | | C | | ***ATP7B*** | NM_000053.3 | | | c.19_20delCA | | | p.Gln7fs | | frameshift_variant | | | rs749363958 | | **Pathogenic** | 0.00015110 | 0 |
| chr19 | | 45357502 | | | C | | T | | ***ERCC2*** | NM_000400.3 | | | c.1349G>A | | | p.Arg450His | | missense_variant | | | rs146632315 | | **Likely Pathogenic** | 0.00014460 | 0 |
| chr1 | | 54871547 | | | C | | T | | ***DHCR24*** | NM_014762.3 | | | c.679G>A | | | p.Ala227Thr | | missense_variant | | | rs201280799 | | **Pathogenic** | 0.00013800 | 0 |
| chr22 | | 41525295 | | | C | | A | | ***ACO2*** | NM_001098.2 | | | c.1708C>A | | | p.Pro570Thr | | missense_variant | | | rs201742113 | | **VUS** | 0.00011170 | 0 |
| chr5 | | 150535808 | | | C | | T | | ***NDST1*** | NM_001543.4 | | | c.1360C>T | | | p.Arg454Cys | | missense_variant | | | rs150009231 | | **VUS** | 0.00010510 | 0 |
| chr6 | | 152133355 | | | C | | T | | ***SYNE1*** | NM_182961.3 | | | c.25922G>A | | | p.Arg8641Gln | | missense_variant | | | rs774776856 | | **VUS** | 0.000123 | 0 |
| chr11 | | 108251029 | | | CAG | | C | | ***ATM*** | NM_000051.3 | | | c.1564_1565delGA | | | p.Glu522fs | | frameshift_variant | | | rs587779817 | | **pathogenic** | 0.00005258 | 0 |
| chr19 | | 43526626 | | | G | | C | | ***ETHE1*** | NM_014297.4 | | | c.115C>G | | | p.Leu39Val | | missense_variant | | | rs201260191 | | **VUS** | 0.00005257 | 0 |
| chr21 | | 32639731 | | | G | | C | | ***SYNJ1*** | NM_003895.3 | | | c.3754C>G | | | p.Arg1252Gly | | missense_variant | | | rs779662077 | | **VUS** | 0.00004602 | 0 |
| chr4 | | 103182843 | | | A | | T | | ***CENPE*** | NM_001813.2 | | | c.882T>A | | | p.Asn294Lys | | missense_variant | | | rs765243925 | | **VUS** | 0.00004600 | 0 |
| chr17 | | 42692631 | | | G | | A | | ***CNTNAP1*** | NM_003632.2 | | | c.2663G>A | | | p.Arg888Gln | | missense_variant | | | rs372141725 | | **VUS** | 0.00004599 | 0 |
| chr20 | | 45953747 | | | T | | C | | ***ZNF335*** | NM_022095.3 | | | c.2644A>G | | | p.Ser882Gly | | missense_variant | | | rs919317191 | | **VUS** | 0.00003943 | 0 |
| chr1 | | 46189498 | | | T | | A | | ***POMGNT1*** | NM_001243766.1 | | | c.1855A>T | | | p.Asn619Tyr | | missense_variant | | | rs374401585 | | **Likely Pathogenic** | 0.00003286 | 0 |
| chr2 | | 237363363 | | | C | | T | | ***COL6A3*** | NM_004369.3 | | | c.5953G>A | | | p.Val1985Met | | missense_variant | | | rs200478135 | | **VUS** | 0.00002629 | 0 |
| chr10 | | 12094171 | | | C | | T | | ***DHTKD1*** | NM_018706.6 | | | c.1258C>T | | | p.Arg420Cys | | missense_variant | | | rs762746645 | | **VUS** | 0.00002629 | 0 |
| chr9 | | 2645604 | | | G | | A | | ***VLDLR*** | NM_003383.4 | | | c.1343G>A | | | p.Arg448Gln | | missense_variant | | | rs137946976 | | **VUS** | 0.00002629 | 0 |
| chr4 | | 84677343 | | | G | | C | | ***WDFY3*** | NM_014991.4 | | | c.10313C>G | | | p.Ser3438Cys | | missense_variant | | | rs781067446 | | **VUS** | 0.00002629 | 0 |
| chr5 | | 149004856 | | | G | | A | | ***SH3TC2*** | NM_024577.3 | | | c.3722C>T | | | p.Ala1241Val | | missense_variant | | | rs141712019 | | **VUS** | 0.00001972 | 0 |
| chr13 | | 110150407 | | | G | | A | | ***COL4A1*** | NM_001845.5 | | | c.4966C>T | | | p.Arg1656Cys | | missense_variant | | | rs377350886 | | **VUS** | 0.00001971 | 0 |
| chr14 | | 77278785 | | | C | | T | | ***POMT2*** | NM_013382.5 | | | c.1976G>A | | | p.Arg659Gln | | missense_variant | | | rs770606360 | | **Likely Pathogenic** | 0.00001315 | 0 |
| chr7 | | 140834797 | | | C | | T | | ***BRAF*** | NM_004333.4 | | | c.316G>A | | | p.Gly106Arg | | missense_variant | | | rs749247588 | | **VUS** | 0.00001314 | 0 |
| chr2 | | 165367366 | | | G | | T | | ***SCN2A*** | NM_001040142.1 | | | c.3670G>T | | | p.Ala1224Ser | | missense_variant | | | rs780330020 | | **VUS** | 0.00001314 | 0 |
| chr9 | | 34649479 | | | C | | T | | ***GALT*** | NM_000155.4 | | | c.974C>T | | | p.Pro325Leu | | missense_variant | | | rs111033794 | | **Pathogenic** | 0.00001190 | 0 |
| chr17 | | 2681755 | | | G | | A | | ***PAFAH1B1*** | NM_000430.3 | | | c.1186G>A | | | p.Val396Ile | | missense_variant | | | rs781058937 | | **Likely Pathogenic** | 0.00001190 | 0 |
| chr2 | | 165994205 | | | T | | A | | ***SCN1A*** | NM_001165963.1 | | | c.4793A>T | | | p.Tyr1598Phe | | missense_variant | | | rs377325221 | | **Pathogenic** | 0.00000799 | 0 |
| chr14 | | 87968335 | | | G | | A | | ***GALC*** | NM_000153.3 | | | c.908C>T | | | p.Ser303Phe | | missense_variant | | | rs756352952 | | **Pathogenic** | 0.00000659 | 0 |
| chr2 | | 232833017 | | | G | | T | | ***GIGYF2*** | NM_001103147.1 | | | c.2753G>T | | | p.Arg918Leu | | missense_variant | | | rs376165544 | | **VUS** | 0.00000658 | 0 |
| chr2 | | 74361555 | | | G | | A | | ***DCTN1*** | NM_004082.4 | | | c.3781C>T | | | p.Arg1261Trp | | missense_variant | | | rs768025820 | | **VUS** | 0.00000658 | 0 |
| chr22 | | 40361282 | | | G | | A | | ***ADSL*** | NM_000026.3 | | | c.802G>A | | | p.Asp268Asn | | missense_variant | | | rs746501563 | | **Pathogenic** | 0.00000657 | 0 |
| chr2 | | 240740133 | | | G | | A | | ***KIF1A*** | NM_001244008.1 | | | c.3826C>T | | | p.Arg1276* | | stop_gained | | | rs368078424 | | **Pathogenic** | 0 | 0 |
| chr7 | | 103535484 | | | G | | A | | ***RELN*** | NM_005045.3 | | | c.7181C>T | | | p.Ala2394Val | | missense_variant | | | rs762035577 | | **Pathogenic** | 0.00000657 | 0 |
| chr20 | | 8628307 | | | G | | A | | ***PLCB1*** | NM_015192.3 | | | c.260G>A | | | p.Arg87His | | missense_variant | | | rs750475493 | | **VUS** | 0.00000657 | 0 |
| chr10 | | 60198399 | | | G | | A | | ***ANK3*** | NM_020987.4 | | | c.1630C>T | | | p.His544Tyr | | missense_variant | | | rs780344506 | | **VUS** | 0.00000657 | 0 |
| chr9 | | 137106155 | | | C | | T | | ***MAN1B1*** | NM_016219.4 | | | c.1285C>T | | | p.His429Tyr | | missense_variant | | | rs769255216 | | **VUS** | 0.00000657 | 0 |
| chr7 | | 100102940 | | | G | | A | | ***AP4M1*** | NM_004722.3 | | | c.331G>A | | | p.Glu111Lys | | missense_variant | | | rs757419197 | | **Likely Pathogenic** | 0.00000398 | 0 |
| chr20 | | 8716287 | | | C | | T | | ***PLCB1*** | NM_015192.3 | | | c.1274C>T | | | p.Ala425Val | | missense_variant | | | rs1171674168 | | **VUS** | 0.00000398 | 0 |
| chr7 | | 103482942 | | | C | | T | | ***RELN*** | NM_005045.3 | | | c.10211G>A | | | p.Arg3404His | | missense_variant | | | rs1473736832 | | **VUS** | 0.00000398 | 0 |
| chr20 | | 63444708 | | | C | | T | | ***KCNQ2*** | NM_172107.4 | | | c.641G>A | | | p.Arg214Gln | | missense_variant | | | rs1057518555 | | **Pathogenic** | 0 | 0 |
| chr2 | | 166052875 | | | A | | G | | ***SCN1A*** | NM_006920.6 | | | c.671T>C | | | p.Leu224Ser | | missense_variant | | | rs796053091 | | **Pathogenic** | 0 | 0 |
| chr2 | | 165163616 | | | A | | C | | ***SCN3A*** | NM_006922.4 | | | c.694+2T>G | | | _ | | splice site_variant | | | rs1574270768 | | **Pathogenic** | 0 | 0 |
| chr12 | | 51762639 | | | A | | T | | ***SCN8A*** | NM_001330260.2 | | | c.2507A>T | | | p.Asp836Val | | missense_variant | | | _ | | **VUS** | 0 | 0 |
| chr6 | | 33443340 | | | CCT | | C | | ***SYNGAP1*** | NM_006772.3 | | | c.2793_2794del | | | p.Phe932fs | | frameshift_variant | | | rs1554122252 | | **Pathogenic** | 0 | 0 |
| chr6 | | 33432724 | | | C | | T | | ***SYNGAP1*** | M_006772.3 | | | c.427C>T | | | p.Arg143* | | stop_gained | | | rs397514741 | | **Pathogenic** | 0 | 0 |
| chr16 | | 2496180 | | | A | | G | | ***TBC1D24*** | NM_020705.3 | | | c.32A>G | | | p.Asp11Gly | | missense_variant | | | _ | | **VUS** | 0 | 0 |

Table 3 Statistics for variants in study group and the thousand polish genomes.

|  |  |  |  | **Odds ratio** | **95% CL:** | **Significance level** |
| --- | --- | --- | --- | --- | --- | --- |
| chr16 | 70265641 | T | C | 6.4966 | 0.5856 to 72.0750 | P = 0.1275 |
| chr10 | 95621105 | C | T | 4.3287 | 0.4474 to 41.8790 | P = 0.2057 |
| chr13 | 51974717 | A | G | 6.4966 | 0.5856 to 72.0750 | P = 0.1275 |
| chr13 | 51961868 | G | A | 3.2448 | 0.3603 to 29.2217 | P = 0.2939 |
| chr11 | 134153147 | C | T | 4.3287 | 0.4474 to 41.8790 | P = 0.2057 |
| chr1 | 26795056 | C | A | 8.8263 | 2.4623 to 31.6379 | P = 0.0008 |
| chr13 | 50945445 | G | A | 4.3287 | 0.4474 to 41.8790 | P = 0.2057 |
| chr6 | 73600406 | A | G | 1.8512 | 0.2262 to 15.1497 | P = 0.5658 |
| chr11 | 6617040 | G | A | 2.5945 | 0.3011 to 22.3560 | P = 0.3856 |
| chr8 | 99720377 | A | T | 6.4966 | 0.5856 to 72.0750 | P = 0.1275 |
| chr8 | 99853957 | A | G | 3.7025 | 0.7622 to 17.9852 | P = 0.1045 |
| chr17 | 623567 | C | T | 6.4966 | 0.5856 to 72.0750 | P = 0.1275 |
| The other 45 variants | | | | 38.8969 | 1.5774 to 959.1246 | P = 0.0252 |

Table 4 Rare mitochondrial variants from 54 Patients.

| **Sample ID** | **Nucleotide Position** | **Locus** | **Nucleotide (AA Change)** | **Variant-Level [%]** | **Odds ratio 95% CL:** | **Significance level** | **Helix Frequency in 195983 seqs** | **Helix base-number homoplasmic vs heteroplasmic** | **Conservation** | **APOGEE** |
| --- | --- | --- | --- | --- | --- | --- | --- | --- | --- | --- |
| Patient XVIII | 3209 | MT-RNR2,MT-RNR3 | A-G (rRNA) | 1.74 | 387.2964 CL: 48.9959 to 3061.4536 | P < 0.0001 | 10 0.005% | 10/1 | 62.22% | NA |
| Patient III | 5521 | MT-TW | G-A (tRNA Trp) | 1 | 4260.4783 CL: 262.4999 to 69149.2529 | P < 0.0001 | 0 0.000% | 0/1 | 93.33% | Pathogenic |
| Patient IX | 5536 | MT-TW | A-G (tRNA) | 1.36 | 2130.2283 CL: 189.8336 to 23904.4706 | P < 0.0001 | 1 0.001% | 1/1 | 93.33% | MitoTIP:24.90% likely benign HmtVar: 0.05 |
| Patient VI | 5591 | MT-TA | G-A (tRNA) | 1 | 4260.4783 CL: 262.4999 to 69149.2529 | P < 0.0001 | 0 0.000% | 0/1 | 91.11% | MitoTIP:68.40% possibly pathogenic HmtVar: 1 |
| Patient XX | 7452 | MT-TS1 | A-G (tRNA) | 1.5 | 12644.0968 CL: 508.4648 to 314423.2767 | P < 0.0001 | NR | 0/0 | 95.56% | MitoTIP:59.80% possibly pathogenic HmtVar:0.05 |
| Patient VII | 7947 | MT-CO2 | A-G (Y121C) | 1.9 | 12644.0968 CL: 508.4648 to 314423.2767 | P < 0.0001 | 0 0.000% | 0/0 | NA | NA |
| Patient XXI | 8463 | MT-ATP8 | A-G (Y33C) | 99 | 76.0586 CL: 10.3096 to 561.1212 | P < 0.0001 | 55 0.028% | 55/1 | 53.33% | P (0.53) possibly pathogenic |
| Patient XXII | 9166 | MT-ATP6 | T-C (F214L) | 1.36 | 224.2151 CL: 29.4009 to 1709.8947 | P < 0.0001 | 19 0.010% | 19/0 | 100.00% | N (0.4) possibly benign |
| Patient VI | 9445 | MT-CO3 | G-A (R80Q) | 0.8 | 387.2964 CL: 48.9959 to 3061.4536 | P < 0.0001 | 0 0.000% | 0/11 | 100.00% | N (0.39) possibly benign |
| Patient XXIII | 10405 | MT-TR | T-C (tRNA) | 2.3 | 1065.1033 CL: 116.8047 to 9712.3266 | P < 0.0001 | 0 0.000% | 0/4 | 77.78% | MitoTIP:61.00% possibly pathogenic HmtVar:0.05 |
| Patient XXIV | 11460 | MT-ND4 | T-C (V234A) | 15.25 | 2130.2283 CL: 189.8336 to 23904.4706 | P < 0.0001 | 1 0.001% | 1/1 | 46.67% | N (0.39) possibly benign |

Table 5 Prediction score for m.7937A>G variant by Mitimpct 3D.

| **Conserved score** | |
| --- | --- |
| PhyloP 100v | 5.83 |
| **Pathogenicity predictors** | 13/16 pathogenic character |
| PolyPhen2 | Probably damaging |
| SIFT | Neutral |
| PROVEAN | Deleterious |
| MutationTaster | Disease causing |
| CADD | Deleterious |
| **Pathogenicity meta-predictors** | 5/5 pathogenic character |
| APOGEE | Pathogenic |
| MtoolBox | Deleterious |


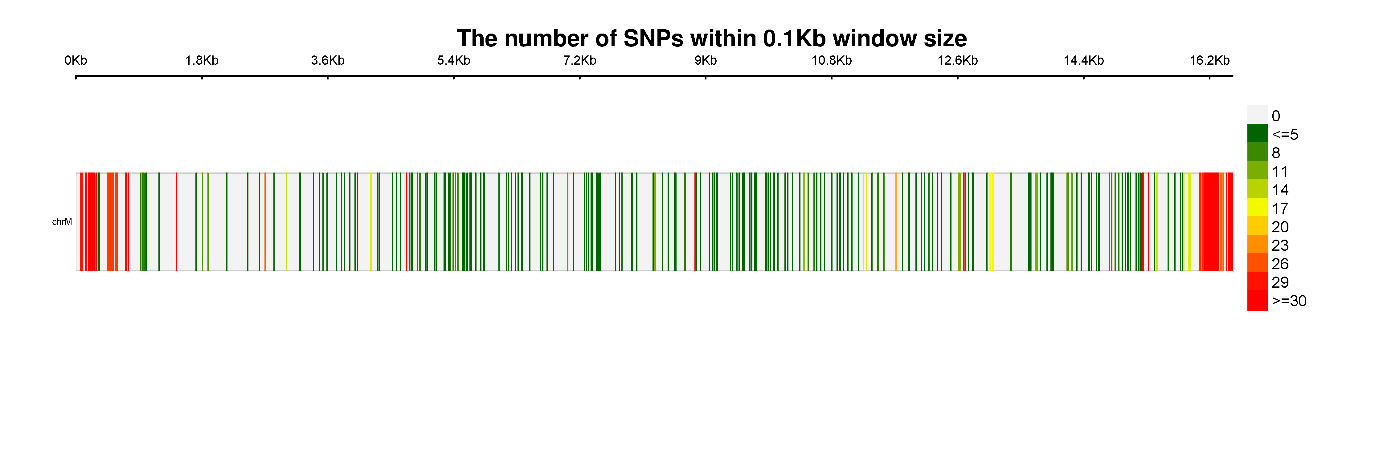


Figure 5 The number of SNPs within 0.1 Kb window size. SNP density was plotted by http://www.bioinformatics.com.cn/plot_basic_SNP_density_by_CMplot_107_en, an online platform for data analysis and visualization.
